# Interpreting missense mutations in Human TRIM5alpha by computational methods

**DOI:** 10.1186/1756-0500-1-116

**Published:** 2008-11-24

**Authors:** Philip A Chan

**Affiliations:** 1 Department of Internal Medicine, Rhode Island Hospital, Providence, RI 02903, USA

## Abstract

**Background:**

The human restriction factor TRIM5α may play an important role in regulation of the human immunodeficiency virus (HIV). It is unclear whether non-synonymous single nucleotide polymorphisms (nsSNP) in TRIM5α affect the clinical course of HIV infection.

**Findings:**

We surveyed the literature for TRIM5α nsSNPs and used comparative sequence analysis to predict the effect of each polymorphism on protein function. Twenty-eight nsSNPs were identified with available functional data, clinical data, or both. The four comparative method programs assessed included SIFT, PolyPhen, A-GVGD, and average BLOSUM62 pairwise score. Two common polymorphisms, H43Y and R136Q, were predicted to be benign based on comparative sequence analysis. The nsSNPs P323R, K324N, I328M, G330Q, R332P, I348V, and T369S were all predicted to affect protein function.

**Conclusion:**

Comparative sequence analysis offers a functional tool to analyze unknown nsSNPs in TRIM5α.

## Background

Human immunodeficiency virus type 1 (HIV-1) infection depends on both viral and human genetic factors [1]. Single nucleotide polymorphisms (SNP) in different immune-modulation genes have been shown to affect susceptibility and progression of disease. Of these, the restriction factors APOBEC3F [2], APOBEC3G [3], and TRIM5α [4] are innate immune proteins that affect postentry steps in HIV-1 replication and confer resistance to retroviruses in other species.

The tripartite motif restriction factor TRIM5α is a cytoplasmic and nucleolic protein that restricts viral infection by interfering with the capsid protein, promoting premature disassembly [5]. TRIM5α has been studied in primates where it has been shown to be extremely effective at inhibiting HIV-1 and other lentiviruses [6,7]. The restriction factor is composed of several regions: the RING domain, B-boxes, a coiled-coil domain, and a carboxy-terminal SPRY (B30.2) domain [8]. The SPRY domain defines antiretroviral activity of TRIM5α and amino acids in this region show a high degree of positive selection based on sequence comparison in primates [9,10]. The variation in the SPRY domain is responsible for the specificity of TRIM5α in primates, but not humans, to effectively restrict HIV-1. The RING domain contributes to the antiviral activity, but the exact function remains unknown [8,10,11]. Different studies have analyzed TRIM5α nsSNPs in both HIV-1 infected and non-infected populations [12-16]. The affect of TRIM5α polymorphisms on protein function and clinical course of HIV-1 remains controversial. Studies have revealed conflicting results secondary to variations in functional assays, lack of power in clinical cohorts, and possible linkage disequilibrium between alleles.

Comparative sequence analysis is a powerful technique that can predict whether an nsSNP is likely to affect protein function. These methods rely on the fact that critical residues for function are conserved across different genomes and should not vary [17-19]. These amino acids may directly participate in enzymatic reaction or have an important role in secondary or tertiary structure. Likewise, residues that are not vital would be subject to increased variation with little to no affect on protein function. Recently, we studied the accuracy of four methods using comparative sequence analysis to predict the affect of nsSNPs on protein function [20]: (1) SIFT (Sorting Intolerant from Tolerant, ) [21]; (2) PolyPhen (Polymorphism Phenotyping, ) [22]; (3) A-GVGD (Grantham Variance-Grantham Difference, ) [19]; (4) Average BLOSUM62 pairwise score [20].

The accuracy of any one method for predicting a non-synonymous SNP as either deleterious (affecting protein function) or tolerant (benign) is approximately 80%. When all four methods agree, the predictive value is greater than 90% [20]. The goals of this study are to: (1) Predict the affect of TRIM5α nsSNPs using comparative sequence analysis and compare our results to known in-vitro and clinical data; (2) Identify mutations that are likely to affect TRIM5α protein function and may warrant further investigation in clinical cohorts and functional assays.

## Methods

### Creation of multiple sequence alignments

Amino acid sequence alignments were constructed using the standard program ClustalW as previously described [18]. Homologs of genes of interest were retrieved from GenBank after BLAST searches using the human sequence as the query. Alignments are available as supplemental material.

The TRIM5α sequence alignment consisted of 40 sequences: *Homo sapiens *(gi 48994821), *Pan paniscus *(gi 122145800), *Pan troglodytes *(gi 60593103), *Gorilla gorilla *(gi 56480705), *Pongo pygmaeus *(gi 122143969), *Pongo abelii *(gi 75060761), *Symphalangus syndactylus *(gi 122143726), *Nomascus leucogenys *(gi 156079722), *Hylobates lar *(gi 122143029), *Colobus guereza *(gi 75060798), *Macaca mulatto *(gi 62548080), *Bunopithecus hoolock *(gi 122144995), *Pygathrix nemaeus *(gi 75060797), *Cercocebus torquatus *(gi 118772044), *Macaca fascicularis *(gi 75060455), *Papio anubis *(gi 162951988), *Macaca assamensis *(gi 122144997), *Macaca nemestrina *(gi 122146076), *Erythrocebus patas *(gi 75060791), *Chlorocebus aethiops *(gi 48994825), *Cercopithecus tantalus *(gi 47559193), *Chlorocebus pygerythrus *(gi 75060767), *Erythrocebus patas *(gi 58379053), *Callithrix jacchus *(gi 167427342), *Callithrix pygmaea *(gi 75060793), *Pithecia pithecia *(gi 75060790), *Saguinus oedipus *(gi 122145799), *Saguinus labiatus *(gi 75060764), *Callicebus donacophilus *(gi 75060786), *Saimiri boliviensis *(gi 58379043), *Saimiri sciureus *(gi 75060788), *Ateles geoffroyi *(gi 75060789), *Lagothrix lagotricha *(gi 75060785), *Aotus trivirgatus *(gi 51317461), *Alouatta sara *(gi 75060794), *Equus caballus *(gi 149719383), *Bos taurus *(gi 77736574), *Mus musculus *(gi 31982207), *Rattus norvegicus *(gi 109459178), and *Gallus gallus *(gi 150247142).

TRIM5α nsSNPs were evaluated by four publicly available methods as previously described [20]: 1) Average BLOSUM62 pairwise; 2) SIFT; 3) PolyPhen; 4) A-GVGD. These computational methods were applied to known clinical and functional data. Literature containing TRIM5α nsSNPs was identified by searching PubMed [12,13,15,16,23]. The agreement of the four methods was assessed for overall consistency using Fleiss' kappa [24,25].

## Results and discussion

Comparative sequence analysis is a powerful tool for the analysis of nsSNPs in the human genome. A large variety of organisms selected for sequence analysis means fewer sequences will be needed to make inferences secondary to long divergence times and increased number of mutations [20,26]. Too little variation can cause residues to be overly conserved with 'false positive' results, i.e. a residue may seem to be critical for protein function when it is not. In our study population, organisms were not highly diversified, but there was sufficient variation for comparative analysis [18,26]. With proper alignment, comparative sequence alignment programs have been shown to be accurate over 90% of the time [20]. The TRIM5α sequence alignment was based on available BLAST data of 40 species with 2550 variants. This met the previous threshold for statistical significance [18,26]. All sequences were eukaryotes and included primates, mouse, rat, and cow [27].

Twenty-eight amino acid mutations in TRIM5α were identified in the literature, twenty-one of which are known to be nsSNPs in the human population (Table 1). Eight other nsSNPs had in-vitro functional data and are located in the critical SPRY domain of TRIM5α, but are not found in humans. The most common nsSNPs found in both HIV-1 infected and uninfected people were H43Y (frequency 6 to 43%) and R136Q (11 to 38%). Other common nsSNPs included V112F (1 to 11%), G249D (6 to 27%), and H419Y (1 to 8%).

**Table 1:** Observed TRIM5α mutations

**nsSNP**	**Domain**	**Observed Frequency**	**In-vitro Functional Assays**	**Affect on Clinical Outcomes**
H43Y	RING	6–43% [12,13,15,16,23]	Functional [12,14,15]; Decreased [13,23]	No effect [12-15]; Accelerated disease progression [16]
C58Y	RING	< 1% [13]	ND	ND
V77A	Linker 1	< 1% [13]	ND	ND
D93V	Linker 1	< 1% [13]	ND	ND
G110E	B-box 2	< 1 to 2% [13,15]	Functional/Slightly decreased [15]	No effect [15]
V112F	B-box 2	1–11% [12,13,15]	Functional [15,23]/Slightly decreased [13]	No effect [12,13,15]
R119W	B-box 2	< 1% [13,15]	Functional [15]	No effect [15]
R119Q	B-box 2	1% [15]	Functional [15]	No effect [15]
R136Q	Coil	11–38% [12,13,15,16]	Functional [12,15,23]/Increased activity [13]	No effect [12,13,16]; Slower progression (in X4 variants) [16]; Increased haplotype frequency in HIV-1 but no affect on disease progression [15]; Suggested protective effect [13]
V140L	Coil	< 1% [13,15]	Functional [15]	No effect [15]
Q143R	Coil	< 1% [13]	ND	ND
R238W	Linker 2	< 5% [23]	Functional [23]	ND
G249D	Linker 2	6–27% [12,13]	Functional [12,13,23]	No effect [13]; Slower disease progression [12]
P323R	SPRY	ND	Functional [10]	ND
K324N	SPRY	ND	Increased activity [10]	ND
I328M	SPRY	ND	Functional [10,29]	ND
G330Q	SPRY	ND	Functional [10,29]	ND
R332P	SPRY	ND	Increased activity [10,29]	ND
I348V	SPRY	ND	Functional [29]	ND
T369S	SPRY	ND	Functional [29]	ND
H419Y	SPRY	1–8% [12,13,15]	Functional [12,13,15,23]	No effect [13,15]; Slower disease progression [12]
V423F	SPRY	< 1% [13]	Functional [13]	ND
V438G	SPRY	< 1% [13]	ND	ND
Y444C	SPRY	< 1% [13]	ND	ND
A446S	SPRY	< 1% [13]	ND	ND
I461L	SPRY	< 1% [13]	Functional [13]	ND
S470P	SPRY	< 1% [13]	Functional [13]	ND
P479L	SPRY	up to 5% [13]	Functional [13]	ND

ND: Not determined

The nsSNP H43Y of the RING region may be important in protein function, specifically E3 ligase activity [13,16,23]. Up to 43% of certain populations carry this polymorphism [23]. Functional data have shown that H43Y retains restriction activity [12,14,15], whereas other results show decreased activity [13,23]. Individuals homozygous for the H43Y mutation may develop X4-trophic virus more rapidly than those who are not and progress to AIDS at a faster rate [16]. To further investigate the affect of H43Y and other polymorphisms on protein function, the twenty-eight TRIM5α mutations were analyzed using SIFT, PolyPhen, A-GVGD, and average BLOSUM62 pairwise score (Table 2). Three of the four computational methods (SIFT, PolyPhen, A-GVGD) suggested that H43Y is a tolerated mutation and does not affect protein function. Although PolyPhen and SIFT do not require aligned sequences, we have previously shown that using a sequence alignment of curated data is superior to a single query sequence alone [20]. In this case, regardless of the sequence(s) entered, SIFT classifies the mutation as tolerant. The BLOSUM62 pairwise program predicted H43Y as deleterious, but does not distinguish specific mutations at a given codon and instead makes general predictions based on overall conservation at a given position. This may be a less specific algorithm for detecting individual mutations, but is still as accurate as the other methods. For H43Y, the agreement between programs suggests a greater than 70% accuracy of a 'tolerant' prediction [20]. This supports the evidence that H43Y does not affect TRIM5α function and is likely a benign mutation.

**Table 2:** TRIM5α mutations and predicted affect on protein function

**nsSNP**	**PolyPhen**	**SIFT**^†^	**A-GVGD**	**B62PW**	**Prediction**^‡^
H43Y	BENIGN	TOLERATED	NEUTRAL	DELETERIOUS	Likely Tolerated (P_i _= 0.50)
C58Y	PROBABLY DAMAGING	DELETERIOUS	DELETERIOUS	DELETERIOUS	Deleterious (P_i _= 1.00)
V77A	BENIGN	TOLERATED	NEUTRAL	TOLERATED	Tolerated (P_i _= 1.00)
D93V	BENIGN	DELETERIOUS	NEUTRAL	DELETERIOUS	Unknown (P_i _= 0.33)
G110E	BENIGN	TOLERATED	NEUTRAL	TOLERATED	Tolerated (P_i _= 1.00)
V112F	POSSIBLY DAMAGING	DELETERIOUS	NEUTRAL	TOLERATED	Unknown (P_i _= 0.33)
R119W	PROBABLY DAMAGING	DELETERIOUS	DELETERIOUS	DELETERIOUS	Deleterious (P_i _= 1.00)
R119Q	BENIGN	DELETERIOUS	POSSIBLY DAMAGING	DELETERIOUS	Likely Deleterious (P_i _= 0.50)
R136Q	BENIGN	TOLERATED	NEUTRAL	TOLERATED	Tolerated (P_i _= 1.00)
V140L	BENIGN	TOLERATED	NEUTRAL	TOLERATED	Tolerated (P_i _= 1.00)
Q143R	POSSIBLY DAMAGING	DELETERIOUS	POSSIBLY DAMAGING	DELETERIOUS	Deleterious (P_i _= 1.00)
R238W	PROBABLY DAMAGING	DELETERIOUS	POSSIBLY DAMAGING	DELETERIOUS	Deleterious (P_i _= 1.00)
G249D	BENIGN	TOLERATED	NEUTRAL	DELETERIOUS	Likely Tolerated (P_i _= 0.50)
P323R	BENIGN	TOLERATED	NEUTRAL	TOLERATED	Tolerated (P_i _= 1.00)
K324N	BENIGN	TOLERATED	NEUTRAL	TOLERATED	Tolerated (P_i _= 1.00)
I328M	BENIGN	TOLERATED	NEUTRAL	TOLERATED	Tolerated (P_i _= 1.00)
G330Q	BENIGN	TOLERATED	NEUTRAL	TOLERATED	Tolerated (P_i _= 1.00)
R332P	BENIGN	TOLERATED	NEUTRAL	TOLERATED	Tolerated (P_i _= 1.00)
I348V	BENIGN	TOLERATED	NEUTRAL	DELETERIOUS	Likely Tolerated (P_i _= 0.50)
T369S	BENIGN	TOLERATED	NEUTRAL	TOLERATED	Tolerated (P_i _= 1.00)
H419Y	BENIGN	TOLERATED	NEUTRAL	TOLERATED	Tolerated (P_i _= 1.00)
V423F	BENIGN	DELETERIOUS	NEUTRAL	TOLERATED	Likely Tolerated (P_i _= 0.50)
V438G	PROBABLY DAMAGING	DELETERIOUS	DELETERIOUS	DELETERIOUS	Deleterious (P_i _= 1.00)
Y444C	BENIGN	DELETERIOUS	NEUTRAL	DELETERIOUS	Unknown (P_i _= 0.33)
A446S	POSSIBLY DAMAGING	DELETERIOUS	NEUTRAL	DELETERIOUS	Likely Deleterious (P_i _= 0.50)
I461L	BENIGN	TOLERATED	NEUTRAL	DELETERIOUS	Likely Tolerated (P_i _= 0.50)
S470P	BENIGN	TOLERATED	NEUTRAL	TOLERATED	Tolerated (P_i _= 1.00)
P479L	PROBABLY DAMAGING	DELETERIOUS	NEUTRAL	DELETERIOUS	Likely Deleterious (P_i _= 0.50)

^†^SIFT output is based on manually aligned sequences. Query only sequence data is not shown.

^‡^Predictions are based on P_i_, the extent to which SIFT (aligned), PolyPhen, A-GVGD, and BLOSUM62 pairwise agree for a given nsSNP. When all four methods agree (P_i _= 1.00), the overall predictive value exceeds 90% [20].

Similar to H43Y, data regarding R136Q has shown conflicting results (Table 1). This amino-acid resides in the coiled coil domain and may participate in TRIM5α oligomerization [8,13]. Clinical studies have shown that this mutation is increased in HIV-infected patients versus non-infected (OR = 5.49, 95% CI 1.83–16.45, p = 0.002) [15], but in-vitro data shows R136Q retains functional activity [15,23]. Furthermore, other clinical studies have shown that R136Q may have a protective affect against HIV-1 [13]. One reason for the conflicting results may be that this mutation is in linkage disequilibrium with other alleles that do play a role in HIV progression or susceptibility [15,16]. A questionable protective effect of R136Q has also been observed in people with X4-trophic virus [16]. Comparative sequence analysis shows that all four methods agree R136Q would be tolerant with an accuracy of greater than 90% [20].

Two other nsSNPs, G249D and H419Y, have also shown ambiguous data with either no effect on clinical outcomes [13,15] or a slower progression of disease [12]. Functional data show that both of these nsSNPs have no affect on TRIM5α function [12,13,15,23]. Three of four methods using comparative sequence analysis suggest G249D is a benign polymorphism, and all four agree that H419 is benign (Table 2).

Several other TRIM5α nsSNPs of interest were also identified. The polymorphisms C58Y, R119W, Q143R, R238W, and V438G are all observed in different human populations and are all predicted deleterious by the four comparative sequence methods (Table 2). Only R119W has been evaluated in clinical studies and has no effect on HIV outcomes [15]. Both R119W and R238W are functional based on in-vitro studies [15,23]. Given the conflicting data, these nsSNPs along with C58Y, Q143R, and V438G should be further studied to assess affect on protein function and association with clinical HIV disease.

The SPRY region of TRIM5α is a critical region involved in species-specific restriction of HIV-1 [8,10,28] and contains codons under high degrees of positive selection [9,28]. A number of TRIM5α mutations have been studied in the SPRY region of the protein (Table 1). Of interest, amino acid residues 325 to 344 are in a segment of this domain which differs from primates [8]. This 'hypervariable' region has been shown to be responsible, at least in part, for the ability to specifically target HIV-1 [10]. Although no nsSNPs have yet been observed in this region in the human population, mutations at this site may confer a protective benefit against HIV-1 infection. In-vitro studies have demonstrated that single amino acid changes in this region, specifically R332P and to a lesser extent K324N, may be able to effectively restrict HIV-1 [10,29]. All methods for these mutations with the exception of I348V were predicted tolerant mutations in agreement with the functional data. For I348V, three methods predicted the mutation as tolerant while only the average BLOSUM62 pairwise predicted it as deleterious.

Overall, the four computational methods agreed the majority of the time (κ = 0.53, moderate agreement [25]). Clinical studies on TRIM5α nsSNPs have shown conflicting results [12,13,15,16], but in-vitro assays clearly demonstrate activity at inhibiting HIV-1. More studies are needed to define the interaction of TRIM5α with immune regulatory genes and DNA sequences that may be in linkage disequilibrium. Focus should be taken to explore the possibility that TRIM5α may affect certain populations differently, specifically people with X4-dominant HIV infection or other ethnic groups.

Two limitations to this study are the paucity of TRIM5α gene sequences available and the lack of structural data available on TRIM5α. Although the number of sequences is sufficient as discussed above, a greater variety of species would allow better alignments based on sensitivity and specificity plots [20]. Furthermore, programs such as PolyPhen rely on structural databases of which there is none presently for TRIM5α.

## Conclusion

Comparative sequence analysis suggests that neither H43Y nor R136Q affect TRIM5α protein function. We identified other nsSNPs that may affect TRIM5α activity and should be analyzed in further clinical and laboratory studies.

## Competing interests

The author declares that they have no competing interests.

## Authors' contributions

PC was the sole contributor to the contents of this article.
